# Exponentiated Odd Lomax Exponential distribution with application to COVID-19 death cases of Nepal

**DOI:** 10.1371/journal.pone.0269450

**Published:** 2022-06-03

**Authors:** Govinda Prasad Dhungana, Vijay Kumar

**Affiliations:** 1 Department of Mathematics and Statistics, Deen Dayal Upadhyaya Gorakhpur University, Gorakhpur, India; 2 Department of Statistics, Tribhuvan University, Birendra Multiple Campus, Bharatpur, Nepal; 3 Deen Dayal Upadhyaya Gorakhpur University, Gorakhpur, India; Stanford University School of Medicine, UNITED STATES

## Abstract

This study suggested a new four-parameter Exponentiated Odd Lomax Exponential (EOLE) distribution by compounding an exponentiated odd function with Lomax distribution as a generator. The proposed model is unimodal and positively skewed whereas the hazard rate function is monotonically increasing and inverted bathtubs. Some important properties of the new distribution are derived such as quintile function and median; asymptotic properties and mode; moments; mean residual life, mean path time; mean deviation; order statistics; and Bonferroni & Lorenz curve. The value of the parameters is obtained from the maximum likelihood estimation, least-square estimation, and Cramér-Von-Mises methods. Here, a simulation study and two real data sets, “the number of deaths per day due to COVID-19 of the first wave in Nepal" and ‘‘failure stresses (In Gpa) of single carbon fibers of lengths 50 mm", have been applied to validate the different theoretical findings. The finding of an order of COVID-19 deaths in 153 days in Nepal obey the proposed distribution, it has a significantly positive relationship between the predictive test positive rate and the predictive number of deaths per day. Therefore, the intended model is an alternative model for survival data and lifetime data analysis.

## Introduction

Probability distributions have been used extensively not only in statistics and mathematics, but also in applied sciences, engineering, and life sciences. Thus, the advancement of probability distributions always continues to grow at a fast pace to simulate real-life conditions and analyze real-life data more efficiently. While doing so, this past decade, many generalized distributions being proposed based on different modification methods with more parameters and flexibility than the existing one. However, there are numerous problems to solve and analyze in real data because any classical or standard probability distributions do not address the different data characteristics [1]. Thus, a new family of distributions or distributions has been proposed to generalize several distributions by compounding well-known distributions which provide greater flexibility in modeling as a practical viewpoint [2].

In the literature, a new parametric distribution has been derived by adding a parameter in exponential and Weibull distribution, yielding a new two-parameter exponential and three parameters Weibull distribution [3]. Marshall–Olkin extended Lomax distribution has been derived by extending the Marshall and Olkin family of distributions based on the Lomax distribution [4]. A five-parameter McDonald Lomax distribution has been derived from the Lomax distribution [5]. Likewise, the new sub-models have been formed by using a Lomax distribution as a generator with two additional positive parameters. In this paper, some special models, such as Lomax-normal, Lomax-Weibull, Lomax-logistic, and Lomax-Pareto distributions have been derived [6, 7]. A new distribution has been generalized, then it became the Kumaraswamy-G Poisson distribution, which has three extra positive parameters [8]. The three-parameter power Lomax distribution, which is more flexible than previous Lomax distributions, and it has been derived with decreasing and inverted bathtub hazard rate functions [9]. Moreover, a new two-parameter half Logistic Poisson distribution has been derived, and it expanded into generalized half-logistic Poisson distribution with three parameters. The proposed distribution is increasing, decreasing, upside-down, and bathtub-shaped hazard rate function [10, 11]. Similarly, a three-parameter Kumaraswamy half logistic distribution has been derived from the Kumaraswamy-G family by compounding with half logistic distribution as a baseline distribution [2].

Furthermore, exponentiated Weibull Lomax distribution has been derived from the exponentiated Weibull-G family [12]. The alpha power inverted exponential distribution has been derived from the inverted exponential distribution with alpha as a power. The proposed distribution is more versatile in numerous real data analyses [13]. An odd generalized exponential family has been compounding with inverted Lomax distribution in modeling, formed four-parameter model is an odd generalized exponentiated inverse Lomax distribution [14]. Likewise, the odd Lomax-exponential (type III) distribution has been derived from the Lomax random variable as a generator [15]. Lomax exponential distribution has been formed after the new modification of the Lomax distribution which is very flexible in life data modeling with decreasing and increasing hazard shapes (non-monotonic) [16]. Similarly, inverse Lomax as a generator has been used in continuous distributions and formed the inverse Lomax-exponentiated-G family [17]. Moreover, a new Poisson inverted exponential distribution is derived from the Poisson-G family [18]. A three-parameter half logistic Nadarajah-Haghighi extension of exponential (NHE) distribution has been derived by compounding a continuous distribution NHE with half logistic-G family [19], and compounding Rayleigh distribution with exponentiated-G Poisson family by power transformation technique formed exponentiated Rayleigh Poisson distribution [20].

In literature, different distributions have been derived and estimated the parameters by different techniques like as; maximum likelihood estimators, least squares estimators, weighted least squares estimators, percentile estimators, the maximum product spacing estimators, the minimum spacing absolute distance estimators, the minimum spacing absolute log-distance estimators, Cramér von Mises estimators, Anderson Darling estimators, right-tailed Anderson Darling estimators, method of moments estimators and Bayes estimators [21–25].

Corona Virus Disease 2019 (COVID-19) pandemic has devastated the world and is accompanied by economic, social, and behavioral challenges and responses. More than 1.5 million people have died worldwide and more than 1,800 people have died in Nepal by the end of December 2020 [26, 27]. Already, several mathematical and statistical models have been proposed to explain the path of the pandemic. However, it is important to note that the characteristics of the data fluctuate which may lead to classical probability distributions that may not be able to be captured in all cases. For example, the data are highly skewed, either to the right or to the left, with the possibility of some outlying observations, and therefore a classical distribution such as the normal distribution cannot be used to fit them. Therefore, flexible distribution is required to capture such data. As a result, we have proposed an Exponentiated Odd Lomax Exponential (EOLE) distribution to analyze the deaths cases of COVID-19 first wave in Nepal. It is more flexible, with four parameters, better equipped to handle complex data, and thus achieves our goal.

In this study, the cumulative distribution function, probability density function, reliability/survival, hazard rate functions, reverse hazard rate function, and cumulative hazard rate function are explicitly presented in section material and methods. Likewise, we derive some important statistical properties such as quintile function and median, asymptotic properties and mode, moments, mean residual life, mean path time, mean deviation, order statistics, and Bonferroni & Lorenz curve. In an estimation technique, we have to employ three well-known estimation methods to estimate the model parameters namely, the Maximum Likelihood Estimation (MLE), Least-Square Estimation (LSE), and Cramér-Von-Mises (CVM). We conducted a simulation study in the result and discussion section, and two real data sets were used to verify the theoretical findings in various aspects. Finally, derive our conclusion of this study with further discussion.

## Materials and methods

### Exponentiated odd Lomax exponential distribution

Exponential distribution plays a significant role in statistics and probability theory. In this distribution, events occur continuously and independently at a constant average rate. It is a special case of gamma, Weibull, Rayleigh, and Erlang distribution. It is a continuous analog of the geometric distribution, which has the main property of being memoryless. As a result, the exponential distribution is used as a baseline probability distribution having a cumulative distribution function

$$G(x)=1-e^{-\alpha x};x>0,\alpha >0.$$ (1)

We have, $\bar{G}(x)=1-G(x);\bar{G}(x)=e^{-\alpha x}$ and $\frac{G(x)}{\bar{G}(x)}=e^{\alpha x}-1$.

The distribution is extended by an auxiliary parameter, it forms an exponentiated function [28, 29]. Let, *θ* > 0 is an auxiliary parameter on odd function, called the exponentiated odd function, which is $W(x)={\left[\frac{G(x)}{\bar{G}(x)}\right]}^{\theta }={\left(e^{\alpha x}-1\right)}^{\theta }$. Similarly, the T-X family of distribution is an extended form of beta generated distribution by taking any non-negative continuous random variable T as a generator instead of beta random variable [30], which is

$$F\left(x\right)=\int_{a}^{W(x)}r\left(t\right)dt$$ (2)

The *r*(*t*) as a generator that has used the probability density function of the Lomax distribution. The Lomax distribution (also known as Pareto type II distribution) is a widespread distribution with applications in the field of actuarial science, reliability modeling, life testing, economics, network analysis, and operations research [31]. Therefore, The PDF of Lomax distribution as a generator is

$$r\left(t\right)=\frac{\lambda }{\delta }{\left\{1+\left(\frac{t}{\delta }\right)\right\}}^{-\left(\lambda +1\right)};\text{t}>0,\lambda >0,\delta >0.$$

We compound the PDF of the Lomax distribution as a generator and exponentiated odd [*W*(*x*)] function because the exponential distribution has a single scale parameter and the Lomax distribution has one of each scale and shape parameter. When both functions are compounded, it becomes two of each scale and shape parameter, making it is more robust and flexible distribution. As a result, it captures different types of data such as; skewed, truncated, non-truncated, and others. Therefore, the CDF of an exponentiated odd Lomax exponential distribution is

$$F\left(x;\alpha ,\lambda ,\theta ,\delta \right)=\int_{0}^{{\left(e^{\alpha x}-1\right)}^{\theta }}\frac{\lambda }{\delta }{\left\{1+\left(\frac{t}{\delta }\right)\right\}}^{-\left(\lambda +1\right)}dt=1-{\left[1+\frac{1}{\delta }{\left\{e^{\alpha x}-1\right\}}^{\theta }\right]}^{-\lambda }.$$ (3)

The corresponding PDF of the proposed distribution is

$$\begin{array}{l} f(x)=\frac{\alpha \theta \lambda }{\delta }e^{\alpha x}{\left(e^{ax}-1\right)}^{\theta -1}{\left[1+\frac{1}{\delta }{\left\{e^{\alpha x}-1\right\}}^{\theta }\right]}^{-(\lambda +1)}\\ \;;x\geq 0,\alpha >0,\lambda >0,\theta >0,\delta >0. \end{array}$$ (4)

**Fig 1:**
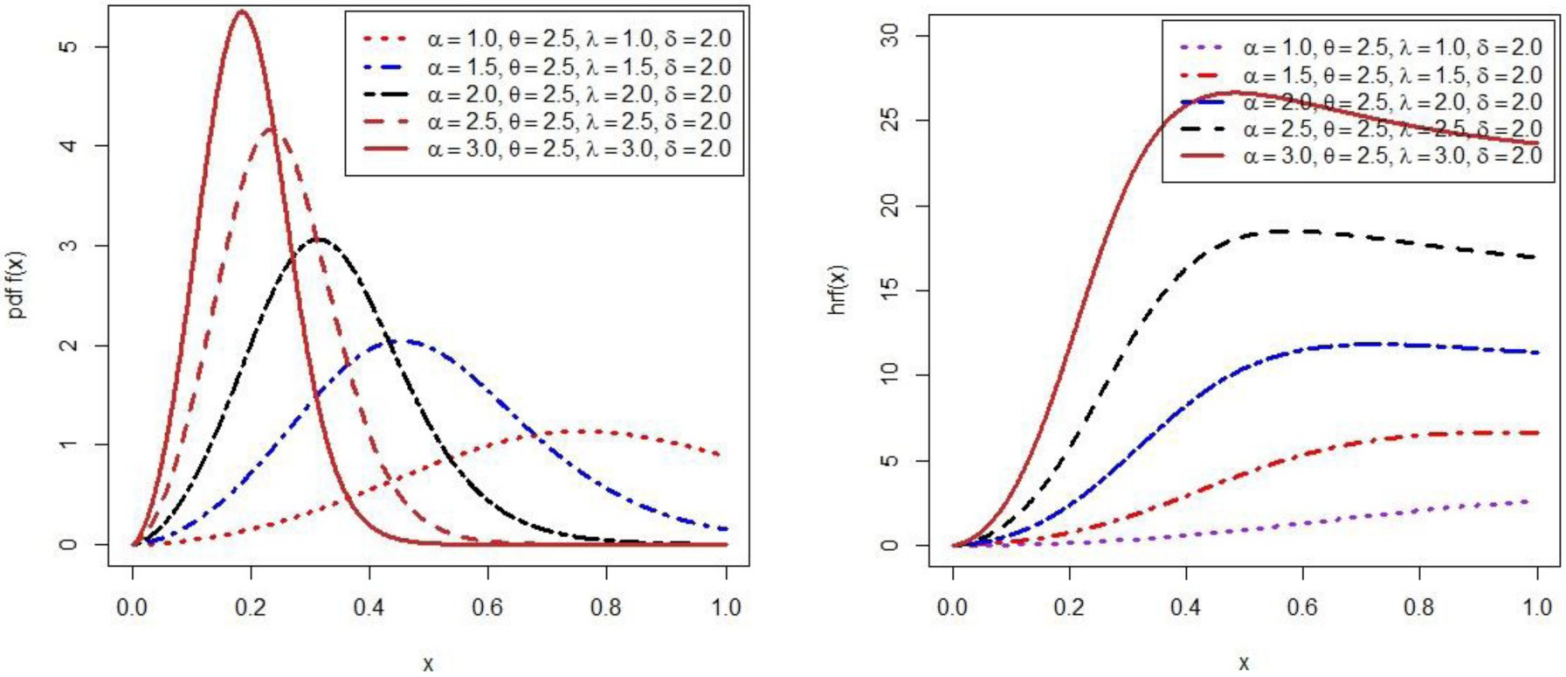
Probability density function (left panel), hazard rate function (right panel) with different parameter’s value.

Here, *α* > 0, *δ* > 0 are scale parameters and *λ* > 0, *θ* > 0 are shape parameters. The shape of PDF (4) is platykurtic and positively skewed at *α* = 1.0, *λ* = 1.0 and *α* = 1.5, *λ* = 1.5, symmetrical at *α* = 2.0, *λ* = 2.0 and it is leptokurtic after increase *α* and *λ* whereas *θ* = 2.5 and *δ* = 2.0 are fixed [Fig 1, (left panel)].

Likewise, the survival function is complementary to the CDF which gives the chance to live just before during ‘*x*’. Mathematically, *R*(*x*) = 1 − *F*(*x*). Hence, the survival function of the proposed distribution is

$$R(x)={\left[1+\frac{1}{\delta }{\left\{e^{\alpha x}-1\right\}}^{\theta }\right]}^{-\lambda }.$$ (5)

The hazard rate function is the conditional density given that the event has not yet occurred before time *x*. Mathematically, let *x* be a survival time of a component or item and we want to calculate the probability that it will not survive for an additional time Δ*x*, then hazard rate function is, $h(x)=\underset{\Delta x\to 0}{\text{lim}}\frac{Prob(x\leq X\leq \Delta x)}{\Delta x.R(x)}=\frac{f(x)}{1-F(x)};x>0.$ Therefore, the hazard rate function of the proposed model is

$$h(x)=\frac{\alpha \theta \lambda }{\delta }e^{\alpha x}{\left(e^{\alpha x}-1\right)}^{\theta -1}{\left[1+\frac{1}{\delta }{\left\{e^{\alpha x}-1\right\}}^{\theta }\right]}^{-1}.$$ (6)

Likewise, the shape of hazard function (6) a is monotonic increase at (*α* = 1.0, *λ* = 1.0), (*α* = 1.5, *λ* = 1.5) and (*α* = 2.0, *λ* = 2.0). After increasing the value of *α* and *λ* then it change monotonic increase and inverted bathtub shaped at (*α* = 2.5, *λ* = 2.5) and (*α* = 3.0, *λ* = 3.0) whereas *θ* = 2.5 and *δ* = 2.0 are fixed [Fig 1, (right panel)].

Similarly, the reversed hazard rate function is the ratio of density to the distribution function which is useful in reliability analysis. It is

$$r(x)=\frac{f(x)}{F(x)}=\frac{\alpha \theta \lambda \frac{1}{\delta }e^{\alpha x}{\left(e^{\alpha x}-1\right)}^{\theta -1}{\left[1+\frac{1}{\delta }{\left\{e^{\alpha x}-1\right\}}^{\theta }\right]}^{-(\lambda +1)}}{1-{\left[1+\frac{1}{\delta }{\left\{e^{\alpha x}-1\right\}}^{\theta }\right]}^{-\lambda }}.$$ (7)

Likewise, the cumulative hazard rate function is not the probability function, however, it measures the risk. Therefore, it is defined as

$$H(x)=-\text{ln}\;R(x)=\lambda \;\text{ln}\left[1+\frac{1}{\delta }{\left\{e^{\alpha x}-1\right\}}^{\theta }\right].$$ (8)

## Statistical properties

In this section, some properties of the EOLE distribution have been derived.

### Useful expansions

Distribution is derived from the generalized binomial series. For, |*Z*| < 1, *n* > 0; we have,

$${\left(1+z\right)}^{-n}=\sum_{i=0}^{\infty }{\left(-1\right)}^{i}\left(\begin{array}{c} n+i-1\\ i \end{array}\right){\;\text{z}}^{i};\;\text{and}\;{\left(1-z\right)}^{n}=\sum_{j=0}^{\infty }{\left(-1\right)}^{j}\left(\begin{array}{c} n\\ j \end{array}\right){\;\text{z}}^{j}.$$ (9)

### Quantile and median

The quantile functions are used in theoretical aspects of a probability distribution. It is an alternative to PDF and CDF, which is used to obtain statistical measures like median, skewness, and kurtosis. It has been also used to generate random numbers. The quantile function is given by *Q*(*u*) = *F*^−1^(*u*). Therefore, the corresponding quantile function of the proposed distribution is

$$Q(u)=\frac{1}{\alpha }\text{ln}\left[1+{\left[\delta \left\{{\left(1-u\right)}^{-\;\frac{1}{\lambda }}-1\right\}\right]}^{\frac{1}{\theta }}\right];0<u<1.$$ (10)

Where, *u* ~ *U*(0,1). In particular, the median is derived by setting $u=\frac{1}{2}$ in Eq (10), we get;

$$\text{Median}=\frac{1}{\alpha }\text{ln}\left[1+{\left[\delta \left\{{\left(\frac{1}{2}\right)}^{-\;\frac{1}{\lambda }}-1\right\}\right]}^{\frac{1}{\theta }}\right].$$

### Asymptotic behavior and mode

To examine the asymptotic behavior, we have to check, $\underset{x\to 0}{lim}f(x)=\underset{x\to \infty }{lim}f(x)$. If both limits are converging into zero, then the proposed model satisfied the properties of asymptotic behavior and it existed the mode value.

Therefore,

$$\underset{x\to 0}{lim}\frac{\alpha \theta \lambda }{\delta }e^{\alpha x}{\left(e^{\alpha x}-1\right)}^{\theta -1}{\left[1+\frac{1}{\delta }{\left\{e^{\alpha x}-1\right\}}^{\theta }\right]}^{-(\lambda +1)}=0.$$

$$\underset{x\to \infty }{lim}\frac{\alpha \theta \lambda }{\delta }e^{\alpha x}{\left(e^{\alpha x}-1\right)}^{\theta -1}{\left[1+\frac{1}{\delta }{\left\{e^{\alpha x}-1\right\}}^{\theta }\right]}^{-(\lambda +1)}=0.$$

Further, we have to calculate the mode by taking the logarithmic in Eq (4), we get;

$$\text{ln}\;f(x)=\text{ln}\left(\frac{\alpha \theta \lambda }{\delta }\right)+\alpha x+(\theta -1)\text{ln}(e^{\alpha x}-1)-(\lambda -1)\text{ln}\left[1+\frac{1}{\delta }{\left\{e^{\alpha x}-1\right\}}^{\theta }\right].$$ (11)

Now, differentiate concerning in Eq (11) and apply the condition *f*(*x*) ≠ 0 and *f*′(*x*) = 0, the mode of proposed distribution is

$$1+\frac{(\theta -1)e^{\alpha x}}{e^{\alpha x}-1}-\frac{\theta (\lambda -1)e^{\alpha x}{\left(e^{\alpha x}-1\right)}^{\theta -1}}{\left\{(e^{\alpha x}-1)+\delta \right\}}=0.$$ (12)

Eq (12) is a nonlinear equation that cannot be solved analytically. It can be solved numerically by using the Newton-Raphson method.

### Moments

The moments of probability distribution suggest the characteristics of the distribution like mean, standard deviation, skewness, and kurtosis. Let, *X* be a random variable following the EOLE distribution, then the moment of the proposed distribution is

$$\mu_{r}^{\prime }=E(X^{r})=\int_{0}^{\infty }x^{r}f(x)dx.$$ (13)

Alternatively, we define the moments of proposed distribution from the quantile function [32, 33]. The *r*^*th*^ raw moment of the proposed distribution is

$$\mu_{r}^{\prime }=\int_{0}^{1}{\left[Q_{G}(u)\right]}^{r}f(x)dx.$$ (14)

Where, *Q*_*G*_(*u*) is the quantile function (10), then Eq (14) is

$$\begin{array}{l} =\int_{0}^{1}{\left[\frac{1}{\alpha }\text{ln}\left[1+{\left\{\delta \left({\left(1-u\right)}^{-\;\frac{1}{\lambda }}-1\right)\right\}}^{\frac{1}{\theta }}\right]\right]}^{r}du\\ =\frac{1}{\alpha^{r}}\int_{0}^{1}{\left[{\left\{\delta \left({\left(1-u\right)}^{-\;\frac{1}{\lambda }}-1\right)\right\}}^{\frac{1}{\theta }}-\frac{1}{2}{\left\{\delta \left({\left(1-u\right)}^{-\;\frac{1}{\lambda }}-1\right)\right\}}^{\frac{2}{\theta }}+\frac{1}{3}{\left\{\delta \left({\left(1-u\right)}^{-\;\frac{1}{\lambda }}-1\right)\right\}}^{\frac{3}{\theta }}+\ldots \right]}^{r}du\\ =\frac{1}{\alpha^{r}}\int_{0}^{1}\delta^{\frac{r}{\theta }}{\left\{{\left(1-u\right)}^{-\;\frac{1}{\lambda }}-1\right\}}^{\frac{r}{\theta }}\sum_{p=0}^{\infty }\alpha_{p}(r){\left\{\delta \left({\left(1-u\right)}^{-\;\frac{1}{\lambda }}-1\right)\right\}}^{\frac{p}{\theta }}du. \end{array}$$ (15)

By simplification, we get *r*^*th*^ raw moments of proposed distribution is

$$=\frac{1}{\alpha^{r}}\sum_{l=0}^{\infty }\sum_{p=0}^{\infty }\sum_{q=0}^{\infty }\frac{{\left(-1\right)}^{q}\alpha_{p}(r)}{l+1}\;(-\delta {)}^{\frac{p+r}{\theta }}\left(\begin{array}{c} \frac{p+r}{\theta }\\ q \end{array}\right)\left(\begin{array}{c} \frac{q}{\lambda }+l-1\\ l \end{array}\right).$$ (16)

Where, *α*_*p*_(*r*) is the coefficient of ${\left\{\delta \left({\left(1-u\right)}^{-\;\frac{1}{\lambda }}-1\right)\right\}}^{\frac{p}{\theta }}$ in the expansion of ${\left\{\sum_{i=1}^{\infty }{\left\{\frac{\delta }{i}\left({\left(1-u\right)}^{-\;\frac{1}{\lambda }}-1\right)\right\}}^{\frac{i}{\theta }}\right\}}^{r}$ [33, 34].

In particular, the first four moments of *X* obtained by substituting the value of *r* = 1, 2, 3 and 4 in Eq (16).


$$E(X)=\frac{1}{\alpha }\sum_{l=0}^{\infty }\sum_{p=0}^{\infty }\sum_{q=0}^{\infty }\frac{{\left(-1\right)}^{q}\alpha_{p}(1)}{l+1}\;(-\delta {)}^{\frac{p+1}{\theta }}\left(\begin{array}{c} \frac{p+1}{\theta }\\ q \end{array}\right)\left(\begin{array}{c} \frac{q}{\lambda }+l-1\\ l \end{array}\right).$$

$$E(X^{2})=\frac{1}{\alpha^{2}}\sum_{l=0}^{\infty }\sum_{p=0}^{\infty }\sum_{q=0}^{\infty }\frac{{\left(-1\right)}^{q}\alpha_{p}(2)}{l+1}\;(-\delta {)}^{\frac{p+2}{\theta }}\left(\begin{array}{c} \frac{p+2}{\theta }\\ q \end{array}\right)\left(\begin{array}{c} \frac{q}{\lambda }+l-1\\ l \end{array}\right).$$

$$E(X^{3})=\frac{1}{\alpha^{3}}\sum_{l=0}^{\infty }\sum_{p=0}^{\infty }\sum_{q=0}^{\infty }\frac{{\left(-1\right)}^{q}\alpha_{p}(3)}{l+1}\;(-\delta {)}^{\frac{p+3}{\theta }}\left(\begin{array}{c} \frac{p+3}{\theta }\\ q \end{array}\right)\left(\begin{array}{c} \frac{q}{\lambda }+l-1\\ l \end{array}\right).$$

$$E(X^{4})=\frac{1}{\alpha^{4}}\sum_{l=0}^{\infty }\sum_{p=0}^{\infty }\sum_{q=0}^{\infty }\frac{{\left(-1\right)}^{q}\alpha_{p}(4)}{l+1}\;(-\delta {)}^{\frac{p+4}{\theta }}\left(\begin{array}{c} \frac{p+4}{\theta }\\ q \end{array}\right)\left(\begin{array}{c} \frac{q}{\lambda }+l-1\\ l \end{array}\right).$$

### Conditional moments

The conditional moment is also of interesting for increasing the failure rate model. Conditional moment is

$$E\left(X^{r}/X>x\right)=\frac{1}{R(x)}\int_{t}^{\infty }x^{r}f(x)dx.$$ (17)

Alternatively, we can define the conditional moments from the quantile function, which is

$$E\left(X^{r}/X>x\right)=\frac{1}{R(x)}\int_{u}^{1}Q{\left(u\right)}^{r}du.$$ (18)

Where, *u* = *F*(*x*) is CDF and, *R*(*x*) is survival function of the proposed model, then conditional moments is

$$E\left(X^{r}/X>x\right)=\frac{1}{\alpha^{r}R(x)}\sum_{l=0}^{\infty }\sum_{p=0}^{\infty }\sum_{q=0}^{\infty }\frac{{\left(-1\right)}^{q}\alpha_{p}(r){\left(-\delta \right)}^{\frac{p+r}{\theta }}}{l+1}\;\left(\begin{array}{c} \frac{p+r}{\theta }\\ q \end{array}\right)\left(\begin{array}{c} \frac{q}{\lambda }+l-1\\ l \end{array}\right)\left[1-{\left\{\text{F}(\text{x})\right\}}^{\left(l+1\right)}\right].$$

In particular,

$$E\left(X/X>x\right)=\frac{1}{\alpha R(x)}\sum_{l=0}^{\infty }\sum_{p=0}^{\infty }\sum_{q=0}^{\infty }\frac{{\left(-1\right)}^{q}\alpha_{p}(1){\left(-\delta \right)}^{\frac{p+1}{\theta }}}{l+1}\;\left(\begin{array}{c} \frac{p+1}{\theta }\\ q \end{array}\right)\left(\begin{array}{c} \frac{q}{\lambda }+l-1\\ l \end{array}\right)\left[1-{\left\{\text{F}(\text{x})\right\}}^{\left(l+1\right)}\right],$$

$$E\left(X^{2}/X>x\right)=\frac{1}{\alpha^{2}R(x)}\sum_{l=0}^{\infty }\sum_{p=0}^{\infty }\sum_{q=0}^{\infty }\frac{{\left(-1\right)}^{q}\alpha_{p}(2){\left(-\delta \right)}^{\frac{p+2}{\theta }}}{l+1}\;\left(\begin{array}{c} \frac{p+2}{\theta }\\ q \end{array}\right)\left(\begin{array}{c} \frac{q}{\lambda }+l-1\\ l \end{array}\right)\left[1-{\left\{\text{F}(\text{x})\right\}}^{\left(l+1\right)}\right],$$

$$E\left(X^{3}/X>x\right)=\frac{1}{\alpha^{3}R(x)}\sum_{l=0}^{\infty }\sum_{p=0}^{\infty }\sum_{q=0}^{\infty }\frac{{\left(-1\right)}^{q}\alpha_{p}(3){\left(-\delta \right)}^{\frac{p+3}{\theta }}}{l+1}\;\left(\begin{array}{c} \frac{p+3}{\theta }\\ q \end{array}\right)\left(\begin{array}{c} \frac{q}{\lambda }+l-1\\ l \end{array}\right)\left[1-{\left\{\text{F}(\text{x})\right\}}^{\left(l+1\right)}\right],$$

and

$$E\left(X^{4}/X>x\right)=\frac{1}{\alpha^{4}R(x)}\sum_{l=0}^{\infty }\sum_{p=0}^{\infty }\sum_{q=0}^{\infty }\frac{{\left(-1\right)}^{q}\alpha_{p}(4){\left(-\delta \right)}^{\frac{p+4}{\theta }}}{l+1}\;\left(\begin{array}{c} \frac{p+4}{\theta }\\ q \end{array}\right)\left(\begin{array}{c} \frac{q}{\lambda }+l-1\\ l \end{array}\right)\left[1-{\left\{\text{F}(\text{x})\right\}}^{\left(l+1\right)}\right].$$

### Mean residual life

The Mean Residual Life (MRL) is the average outstanding life, *X* − *x* given that the item has survived to time *x*. Thus, the expected additional lifetime given that a component has survived until the time *x* is called the MRL. It is defined as,

$$\text{m}(x)=E\left[X-x/X>x\right]=\frac{1}{R(x)}\int_{t}^{\infty }xf(x)dx-x.$$ (19)

Alternatively, we can define the MRL of proposed distribution from the quantile function is

$$\text{m}(x)=E\left[X-x/X>x\right]=\frac{1}{R(x)}\int_{u}^{1}Q(u)du-x.$$ (20)

$$=\frac{1}{\alpha R(x)}\sum_{l=0}^{\infty }\sum_{p=0}^{\infty }\sum_{q=0}^{\infty }\frac{{\left(-1\right)}^{q}\alpha_{p}(1){\left(-\delta \right)}^{\frac{p+1}{\theta }}}{l+1}\;\left(\begin{array}{c} \frac{p+1}{\theta }\\ q \end{array}\right)\left(\begin{array}{c} \frac{q}{\lambda }+l-1\\ l \end{array}\right)\left[1-{\left\{\text{F}(\text{x})\right\}}^{\left(l+1\right)}\right]-x.$$

Where, *F*(*x*) is CDF and, *R*(*x*) is survival function of the proposed distribution.

### Mean past lifetime

The mean Past Lifetime (MPL) is the conditional random variable *x* − *X*/*X* ≤ *x*. This showed that the time elapsed from the failure of the component given that its lifetime is less or equal to *x*. It can be calculated as,

$$k(x)=E\left[x-X/X\leq x\right]=\frac{\int_{0}^{t}F(x)dx}{F(x)}=x-\frac{\int_{0}^{t}xf(x)dx}{F(x)}.$$ (21)

It can be alternatively defined from the quantile function, which is

$$k(x)=x-\frac{\int_{0}^{u}Q(u)du}{F(x)}.$$ (22)

$$=x-\frac{1}{\alpha }\sum_{l=0}^{\infty }\sum_{p=0}^{\infty }\sum_{q=0}^{\infty }\frac{{\left(-1\right)}^{q}\alpha_{p}(1){\left(-\delta \right)}^{\frac{p+1}{\theta }}}{l+1}\;\left(\begin{array}{c} \frac{p+1}{\theta }\\ q \end{array}\right)\left(\begin{array}{c} \frac{q}{\lambda }+l-1\\ l \end{array}\right){\left[\text{F}(\text{x})\right]}^{l}.$$

### Mean deviation

The Mean Deviation (MD) from mean and median measures the scatter from the center value either mean or median. The MD is defined as,

$$MD(\mu )=\int_{0}^{\infty }\left|x-\mu \right|f(x)dx\;\text{and},\;\text{M}D({\text{m}}_{d})=\int_{0}^{\infty }\left|x-m_{d}\right|f(x)dx$$ (23)

We obtained *MD*(*μ*) and MD(*m*_*d*_) using the following relationships:

$$\begin{array}{l} MD(\mu )=\int_{0}^{\mu }\left(\mu -x\right)f(x)dx+\int_{\mu }^{\infty }\left(x-\mu \right)f(x)dx\\ =\mu F(\mu )-\int_{0}^{\mu }xf(x)dx-\mu \left[1-F(\mu )\right]+\int_{\mu }^{\infty }xf(x)dx\;∵\;\left[\int_{\mu }^{\infty }f(x)dx=\int_{0}^{\infty }f(x)dx-\int_{0}^{\mu }f(x)dx\right]\\ =2\mu F(\mu )-2\mu +2\int_{\mu }^{\infty }xf(x)dx.\;∵\left[\int_{0}^{\mu }xf(x)dx=\int_{0}^{\infty }xf(x)dx-\int_{\mu }^{\infty }xf(x)dx\right] \end{array}$$ (24)

Likewise, $MD({\text{m}}_{d})=\int_{0}^{{\text{m}}_{d}}\left({\text{m}}_{d}-x\right)f(x)dx+\int_{{\text{m}}_{d}}^{\infty }\left(x-{\text{m}}_{d}\right)f(x)dx$

$$\begin{array}{l} =m_{d}F(m_{d})-\int_{0}^{m_{d}}xf(x)dx-m_{d}\left[1-F(m_{d})\right]+\int_{m_{d}}^{\infty }xf(x)dx\\ =2m_{d}F(m_{d})-\mu -m_{d}+2\int_{m_{d}}^{\infty }xf(x)dx. \end{array}$$ (25)

We have to calculate $\int_{\mu }^{\infty }xf(x)dx$ in terms of quantile function such as $\int_{u}^{1}Q(u)du$.


$$\int_{\mu }^{\infty }xf(x)dx=\frac{1}{\alpha R(\mu )}\sum_{l=0}^{\infty }\sum_{p=0}^{\infty }\sum_{q=0}^{\infty }\frac{{\left(-1\right)}^{q}\alpha_{p}(1){\left(-\delta \right)}^{\frac{p+1}{\theta }}}{l+1}\;\left(\begin{array}{c} \frac{p+1}{\theta }\\ q \end{array}\right)\left(\begin{array}{c} \frac{q}{\lambda }+l-1\\ l \end{array}\right)\left[1-{\left\{\text{F}(\mu )\right\}}^{\left(l+1\right)}\right].$$

Likewise,

$$\int_{m_{d}}^{\infty }xf(x)dx=\frac{1}{\alpha R({\text{m}}_{d})}\sum_{l=0}^{\infty }\sum_{p=0}^{\infty }\sum_{q=0}^{\infty }\frac{{\left(-1\right)}^{q}\alpha_{p}(1){\left(-\delta \right)}^{\frac{p+1}{\theta }}}{l+1}\;\left(\begin{array}{c} \frac{p+1}{\theta }\\ q \end{array}\right)\left(\begin{array}{c} \frac{q}{\lambda }+l-1\\ l \end{array}\right)\left[1-{\left\{\text{F}({\text{m}}_{d})\right\}}^{\left(l+1\right)}\right].$$

Finally, the Eqs (24) and (25) becomes,

$$\begin{array}{ll} MD(\mu ) & =2\mu F(\mu )-2\mu +2\frac{1}{\alpha R(\mu )}\sum_{l=0}^{\infty }\sum_{p=0}^{\infty }\sum_{q=0}^{\infty }\frac{{\left(-1\right)}^{q}\alpha_{p}(1){\left(-\delta \right)}^{\frac{p+1}{\theta }}}{l+1}\;\left(\begin{array}{c} \frac{p+1}{\theta }\\ q \end{array}\right)\\ & \left(\begin{array}{c} \frac{q}{\lambda }+l-1\\ l \end{array}\right)\left[1-{\left\{\text{F}(\mu )\right\}}^{\left(l+1\right)}\right]. \end{array}$$

and

$$\begin{array}{ll} MD(m_{d}) & =2m_{d}F(m_{d})-\mu -m_{d}+2\frac{1}{\alpha R({\text{m}}_{d})}\sum_{l=0}^{\infty }\sum_{p=0}^{\infty }\sum_{q=0}^{\infty }\frac{{\left(-1\right)}^{q}\alpha_{p}(1){\left(-\delta \right)}^{\frac{p+1}{\theta }}}{l+1}\;\left(\begin{array}{c} \frac{p+1}{\theta }\\ q \end{array}\right)\\ & \left(\begin{array}{c} \frac{q}{\lambda }+l-1\\ l \end{array}\right)\left[1-{\left\{\text{F}({\text{m}}_{d})\right\}}^{\left(l+1\right)}\right]. \end{array}$$

### Order statistics

Order statistics have been extensively applied in many fields of statistics such as reliability and life testing. Let, *X*_1_, *X*_2_, …, *X*_*n*_ random sample from (4) and *X*_1:*n*_ ≤ *X*_2:*n*_ ≤ … ≤ *X*_n:*n*_ corresponding order statistics. The probability density function of *r*^*th*^ order statistics say *X*_*r*:*n*_; 1 ≤ *r* ≤ *n* [33] is given by;

$$f_{r:n}(x)=\frac{n!}{(r-1)!(n-r)!}f(x){\left[F(x)\right]}^{r-1}{\left[1-F(x)\right]}^{n-r}$$ (26)

We apply the preposition of (1) and (2) in Eq (26) then the equation becomes,

$$f_{{}_{r:n}}(x)=\frac{n!}{\left(r-1\right)!\left(n-r\right)!}\sum_{i=0}^{\infty }\sum_{j=0}^{\infty }\Phi_{ij}e^{\alpha (i+j)x_{\left(r\right)}}{\left[1-\sum_{i=0}^{\infty }\sum_{j=0}^{\infty }\eta_{ij}e^{\alpha jx_{\left(r\right)}}\right]}^{r-1}{\left[\sum_{i=0}^{\infty }\sum_{j=0}^{\infty }\eta_{ij}e^{\alpha jx_{\left(r\right)}}\right]}^{n-r}$$ (27)

When, *r* = *n* then from Eq (27), the pdf of the largest order statistics *X*_*n*:*n*_ is given by

$$f_{n:n}(x)=n\sum_{i=0}^{\infty }\sum_{j=0}^{\infty }\Phi_{ij}e^{\alpha (i+j)x_{\left(n\right)}}{\left[1-\sum_{i=0}^{\infty }\sum_{j=0}^{\infty }\eta_{ij}e^{\alpha jx_{\left(n\right)}}\right]}^{n-1};x_{\left(n\right)}>0.$$

Similarly, *r* = 1, then from Eq (27), the pdf of smallest order statistics *x*_1:*n*_ is given by

$$f_{1:n}(x)=n\sum_{i=0}^{\infty }\sum_{j=0}^{\infty }\Phi_{ij}e^{\alpha (i+j)x_{\left(1\right)}}{\left[\sum_{i=0}^{\infty }\sum_{j=0}^{\infty }\eta_{ij}e^{\alpha jx_{\left(1\right)}}\right]}^{n-1};x_{\left(1\right)}>0.$$

### Bonferroni and Lorenz curve

Bonferroni and Lorenz curve has been proposed by Bonferroni [33]. To measure poverty and income, Bonferroni and Lorenz curves are widely used. Also, such types of curves are widely used in other fields like demography, medicine, reliability, insurance and many others.

## Methods of estimation

We have to estimate the value of unknown parameters of the proposed model by maximum likelihood estimation, method of least square, weighted least square, and Cramér von miss technique.

### Maximum Likelihood Estimation (MLE)

Let, *x*_1_, *x*_2_, …, *x*_*n*_ are random sample from EOLE distribution with parameters (*α*, *θ*, *λ* and *δ*), then likelihood function of proposed distribution is the product of *n*^*th*^ time of sample PDF which is $l(x;\underset{˜}{\Phi })=\prod_{i=1}^{n}f(x_{i};\underset{˜}{\Phi })$. Where, $\underset{˜}{\Phi }$ is the parameter space which belongs to (*α*, *θ*, *λ* and *δ*). Therefore, the log-likelihood function of the proposed distribution is

$$\text{ln}(l)=n\;\text{ln}\left(\frac{\alpha \theta \lambda }{\delta }\right)+\alpha \sum_{i=1}^{n}x_{i}+(\theta -1)\sum_{i=1}^{n}\text{ln}\left(e^{\alpha x_{i}}-1\right)-\left(\lambda +1\right)\sum_{i=1}^{n}\text{ln}\left[1+\frac{1}{\delta }{\left(e^{\alpha x_{i}}-1\right)}^{\theta }\right]$$ (28)

The parameters are obtained from maximum likelihood estimation by partial differentiate (28) with respect to corresponding parameters. Let, $\xi_{i}=e^{\alpha x_{i}}$ and $\upsilon_{i}=e^{\alpha x_{i}}-1$ we have;

$$\frac{\partial \text{ln}(l)}{\partial \alpha }=\frac{n}{\alpha }+\sum_{i=1}^{n}x_{i}+\left(\theta -1\right)\sum_{i=1}^{n}\left(\frac{x_{i}\xi_{i}}{\upsilon_{i}}\right)-\left(\lambda +1\right)\theta \sum_{i=1}^{n}\left(\frac{x_{i}\xi_{i}\upsilon_{i}{}^{\theta -1}}{\delta +\upsilon_{i}^{\theta }}\right),$$ (29)

$$\frac{\partial \text{ln}(l)}{\partial \theta }=\frac{n}{\theta }+\sum_{i=1}^{n}\text{ln}(\upsilon_{i})-\left(\lambda +1\right)\sum_{i=1}^{n}\left\{\frac{\upsilon_{i}^{\theta }\text{ln}(\upsilon_{i})}{\left(\delta +\upsilon_{i}^{\theta }\right)}\right\},$$ (30)

$$\frac{\partial \text{ln}(l)}{\partial \lambda }=\frac{n}{\lambda }-\sum_{i=1}^{n}\text{ln}\left(1+\frac{\upsilon_{i}^{\theta }}{\delta }\right),$$ (31)

$$\frac{\partial \text{ln}(l)}{\partial \delta }=-\frac{n}{\delta }+\frac{\left(\lambda +1\right)}{\delta }\sum_{1=1}^{n}\left\{\frac{\upsilon_{i}^{\theta }}{\left(\delta +\upsilon_{i}^{\theta }\right)}\right\}.$$ (32)

Finally, solve non-linear equations $\frac{\partial \text{ln}(l)}{\partial \alpha }=0$, $\frac{\partial \text{ln}(l)}{\partial \beta }=0$, $\frac{\partial \text{ln}(l)}{\partial \lambda }=0$ and $\frac{\partial \text{ln}(l)}{\partial \delta }=0$ for *α*, *θ*, *λ* and *δ*. We get the maximum likelihood estimate value ($\hat{\alpha }$, $\hat{\theta }$, $\hat{\lambda }$ and $\hat{\delta }$) of the parameters (*α*, *θ*, *λ* and *δ*). Likewise, for the interval estimation of parameters (*α*, *θ*, *λ* and *δ*), we have to calculate the observed information matrix. The observed information matrix is

$$I=\left[\begin{array}{cccc} I_{\alpha \alpha } & I_{\alpha \theta } & I_{\alpha \lambda } & I_{\alpha \delta }\\ I_{\alpha \theta } & I_{\theta \theta } & I_{\theta \lambda } & I_{\theta \delta }\\ I_{\alpha \lambda } & I_{\theta \lambda } & I_{\lambda \lambda } & I_{\lambda \delta }\\ I_{\alpha \delta } & I_{\theta \delta } & I_{\lambda \delta } & I_{\delta \delta } \end{array}\right]$$ (33)

The elements of the observed information matrix are in Appendix B of S1 Appendix. Let $\underset{˜}{\Phi }={\left(\alpha ,\theta ,\lambda ,\delta \right)}^{T}$ denote the parameter space and the corresponding MLE of $\underset{˜}{\Phi }$ as $\underset{˜}{\hat{\Phi }}={\left(\hat{\alpha },\hat{\theta },\hat{\lambda },\hat{\delta }\right)}^{T}$, then $\left(\underset{˜}{\hat{\Phi }}-\underset{˜}{\Phi }\right)\to N_{4}\left[0,{\left(I\left(\underset{˜}{\Phi }\right)\right)}^{-1}\right]$ follows the asymptotic multivariate normal distribution, where $I\left(\underset{˜}{\Phi }\right)$ is the Fisher’s information matrix. For practical proposed, we directly calculate the observed information matrix from Eq (28) and convert it into Hussain matrix. Finally, we calculate the variance-covariance matrix from the inverse of the Hussain matrix is

$${\left(-H{\left(\underset{˜}{\Phi }\right)}_{\underset{˜}{\Phi }=\underset{˜}{\hat{\Phi }}}\right)}^{-1}=\left[\begin{array}{cccc} \text{var}(\hat{\alpha }) & \text{cov}(\hat{\alpha },\hat{\theta }) & \text{cov}(\hat{\alpha },\hat{\lambda }) & \text{cov}(\hat{\alpha },\hat{\delta })\\ \text{cov}(\hat{\alpha },\hat{\theta }) & \text{var}(\hat{\theta }) & \text{cov}(\hat{\theta },\hat{\lambda }) & \text{cov}(\hat{\theta },\hat{\delta })\\ \text{cov}(\hat{\alpha },\hat{\lambda }) & \text{cov}(\hat{\theta },\hat{\lambda }) & \text{var}(\hat{\lambda }) & \text{cov}(\hat{\lambda },\hat{\delta })\\ \text{cov}(\hat{\alpha },\hat{\delta }) & \text{cov}(\hat{\theta },\hat{\delta }) & \text{cov}(\hat{\lambda },\hat{\delta }) & \text{var}(\hat{\delta }) \end{array}\right]$$ (34)

Furthermore, the asymptotic normality of MLEs, approximate 100(1 − *γ*)% confidence intervals of *α*, *θ*, *λ* and *δ* can be constructed as;

$$\hat{\alpha }\pm z_{\gamma /2}SE(\hat{\alpha }),\;\hat{\theta }\pm z_{\lambda /2}SE(\hat{\theta })\;\hat{\lambda }\pm z_{\gamma /2}SE(\hat{\lambda })\;\text{and}\;\hat{\delta }\pm z_{\gamma /2}SE(\hat{\delta })\;$$

where *z*_*γ*/2_ is the upper percentile of standard normal variate.

### Method of Least-Square Estimation (LSE)

Initially, the least square estimation and weighted least square estimate were introduced to estimate the parameters of beta distribution [35, 36]. This technique has been used to estimate unknown parameters of proposed distribution by minimizing the concerning parameters *α*, *θ*, *λ* and *δ*, which is

$$U\left(x;\alpha ,\theta ,\lambda ,\delta \right)=\sum_{k=1}^{n}{\left[F\left(x_{k}\right)-\frac{k}{n+1}\right]}^{2}$$

$$=\sum_{k=1}^{n}{\left[1-{\left\{1+\frac{1}{\delta }{\left(e^{\alpha x_{k}}-1\right)}^{\theta }\right\}}^{-\lambda }-\frac{k}{n+1}\right]}^{2}.$$ (35)

The parameter’s values are obtained from the least square method by partial differentiation in Eq (35) concerning corresponding parameters.

Let, $\xi_{k}=e^{\alpha x_{k}}$ and $\upsilon_{k}=e^{\alpha x_{k}}-1$, and $\tau_{k}=1+\frac{1}{\delta }{\left(e^{\alpha x_{i}}-1\right)}^{\theta }$, then Eq (35) becomes;

$$\frac{\partial U}{\partial \alpha }=\frac{2\lambda \theta }{\delta }\sum_{k=1}^{n}x_{k}\xi_{k}\upsilon_{k}^{\theta -1}\tau_{k}^{-(\lambda +1)}\left[1-\tau_{k}^{-\lambda }-\frac{k}{n+1}\right],$$ (36)

$$\frac{\partial U}{\partial \theta }=\frac{2\lambda }{\delta }\sum_{k=1}^{n}x_{k}\upsilon_{k}^{\theta }\;\text{ln}(\upsilon_{k})\tau_{k}^{-(\lambda +1)}\left[1-\tau_{k}^{-\lambda }-\frac{k}{n+1}\right],$$ (37)

$$\frac{\partial U}{\partial \lambda }=2\sum_{k=1}^{n}\tau_{k}^{-\lambda }\;\text{ln}\left\{1+\frac{\upsilon_{k}^{\theta }}{\delta }\right\}\left[1-\tau_{k}^{-\lambda }-\frac{k}{n+1}\right],$$ (38)

$$\frac{\partial U}{\partial \delta }=-\frac{2\lambda }{\delta^{2}}\sum_{k=1}^{n}\upsilon_{k}^{\theta }\tau_{k}^{-(\lambda +1)}\left[1-\tau_{k}^{-\lambda }-\frac{k}{n+1}\right].$$ (39)

We solve non-linear equations $\frac{\partial U}{\partial \alpha }=0$, $\frac{\partial U}{\partial \theta }=0$, $\frac{\partial U}{\partial \lambda }=0$ and $\frac{\partial U}{\partial \delta }=0$ to estimate the unknown parameters of the proposed distribution by minimizing the function concerning parameters *α*, *θ*, *λ* and *δ*.

### Weighted least-square estimation

The weighted least-squares estimation is a technique to determine the unknown parameters by minimizing concerning parameters *α*, *θ*, *λ* and *δ* is

$$\begin{array}{l} W\left(X;\alpha ,\theta ,\lambda ,\delta \right)={\sum_{k=1}^{n}w_{k}\left[F\left(x_{k}\right)-\frac{k}{n+1}\right]}^{2},\\ =\sum_{k=1}^{n}\frac{{\left(n+1\right)}^{2}\left(n+2\right)}{k(n-k+1)}{\left[1-{\left\{1+\frac{1}{\delta }{\left(e^{\alpha x_{k}}-1\right)}^{\theta }\right\}}^{-\lambda }-\frac{k}{n+1}\right]}^{2}. \end{array}$$ (40)

Where, $w_{k}=\frac{1}{V(X_{\left(k\right)})}=\frac{{\left(n+1\right)}^{2}\left(n+2\right)}{k\left(n-k+1\right)}$ is the weight for the proposed model. Hence, the weighted least-square estimators of *α*, *θ*, *λ* and *δ* respectively can be obtained by partial differentiate with respect to corresponding parameters in Eq (40) and set the result equal to zero

$$\frac{\partial W}{\partial \alpha }=\frac{2\lambda \theta }{\delta }\sum_{k=1}^{n}x_{k}w_{k}\xi_{k}\upsilon_{k}^{\theta -1}\tau_{k}^{-(\lambda +1)}\left[1-\tau_{k}^{-\lambda }-\frac{k}{n+1}\right],$$ (41)

$$\frac{\partial W}{\partial \theta }=\frac{2\lambda }{\delta }\sum_{k=1}^{n}w_{k}x_{k}\upsilon_{k}^{\theta }\;\text{ln}(\upsilon_{k})\tau_{k}^{-(\lambda +1)}\left[1-\tau_{k}^{-\lambda }-\frac{k}{n+1}\right],$$ (42)

$$\frac{\partial W}{\partial \lambda }=2\sum_{k=1}^{n}w_{k}\tau_{k}^{-\lambda }\;\text{ln}\left\{1+\frac{\upsilon_{k}^{\theta }}{\delta }\right\}\left[1-\tau_{k}^{-\lambda }-\frac{k}{n+1}\right],$$ (43)

$$\frac{\partial W}{\partial \delta }=-\frac{2\lambda }{\delta^{2}}\sum_{k=1}^{n}w_{k}\upsilon_{k}^{\theta }\tau_{k}^{-(\lambda +1)}\left[1-\tau_{k}^{-\lambda }-\frac{k}{n+1}\right].$$ (44)

We solve non-linear equations $\frac{\partial W}{\partial \alpha }=0$, $\frac{\partial W}{\partial \theta }=0$, $\frac{\partial W}{\partial \lambda }=0$ and $\frac{\partial W}{\partial \delta }=0$ to estimate unknown parameters of proposed distribution by minimizing function concerning parameters *α*, *θ*, *λ* and *δ*.

### Method of Cramér-Von-Mises (CVM)

Cramér-von-Mises is minimum distance estimators [36]. It provides empirical evidence that the bias of the estimator is smaller than the other minimum distance estimators. The CVM estimators are achieved and the function has minimized *C*(*α*, *θ*, *λ*, *δ*)

$$\begin{array}{l} C\left(X;\alpha ,\theta ,\lambda ,\delta \right)=\frac{1}{12n}+{\sum_{k=1}^{n}\left[F\left(x_{\text{k}:n}\left|\alpha ,\theta ,\lambda ,\delta \right.\right)-\frac{2k-1}{2n}\right]}^{2},\\ C(x;\alpha ,\theta ,\lambda ,\delta )=\frac{1}{12n}+\sum_{k=1}^{n}{\left[1-{\left\{1+\frac{1}{\delta }{\left(e^{\alpha x_{k}}-1\right)}^{\theta }\right\}}^{-\lambda }-\frac{2k-1}{2n}\right]}^{2}. \end{array}$$ (45)

Cramér-Von-Mises estimators of *α*, *θ*, *λ* and *δ* respectively can be obtained by partial differentiate with respect to corresponding parameters in Eq (45) and set the result equal to zero

$$\frac{\partial C}{\partial \alpha }=\frac{2\lambda \theta }{\delta }\sum_{k=1}^{n}x_{k}\xi_{k}\upsilon_{k}^{\theta -1}\tau_{k}^{-(\lambda +1)}\left[1-\tau_{k}^{-\lambda }-\frac{2k-1}{2n}\right],$$ (46)

$$\frac{\partial C}{\partial \theta }=\frac{2\lambda }{\delta }\sum_{k=1}^{n}x_{k}\upsilon_{k}^{\theta }\;\text{ln}(\upsilon_{k})\tau_{k}^{-(\lambda +1)}\left[1-\tau_{k}^{-\lambda }-\frac{2k-1}{2n}\right],$$ (47)

$$\frac{\partial C}{\partial \lambda }=2\sum_{k=1}^{n}\tau_{k}^{-\lambda }\;\text{ln}\left\{1+\frac{\upsilon_{k}^{\theta }}{\delta }\right\}\left[1-\tau_{k}^{-\lambda }-\frac{2k-1}{2n}\right],$$ (48)

$$\frac{\partial C}{\partial \delta }=-\frac{2\lambda }{\delta^{2}}\sum_{k=1}^{n}\upsilon_{k}^{\theta }\tau_{k}^{-(\lambda +1)}\left[1-\tau_{k}^{-\lambda }-\frac{2k-1}{2n}\right].$$ (49)

We solve non-linear equations $\frac{\partial C}{\partial \alpha }=0$, $\frac{\partial C}{\partial \theta }=0$, $\frac{\partial C}{\partial \lambda }=0$ and $\frac{\partial C}{\partial \delta }=0$ to estimate unknown parameters of proposed distribution by minimizing the function concerning parameters *α*, *θ*, *λ* and *δ*.

## Results and discussion

Data analysis has been done in two-phase. Firstly, we have done a simulation study and secondly, we have done real data analysis. In real data analysis, two data sets have been used to validate the proposed model: (i) The first data set is the number of deaths per day due to the COVID-19 first wave in Nepal. (ii) The second data set is failure stresses (in GPa) of single carbon fibers of lengths 50 mm.

### Simulation study

In a simulation study, we estimate the parameters of the proposed distribution by maximum likelihood estimation. The performance of ML estimators is assessed through their average bias and Mean Square Error (MSEs) for different sample sizes. For the estimation purpose, 10000 random samples of sizes 50, 200, 500, 750 are generated with different combinations of (*α*, *θ*, *λ* and *δ*). The iterative technique is used to estimate the ML parameters of each sample size. We observed that average bias and MSEs for individual parameters fall to zero when sample size increases as our expectation, which provides the consistency of the estimators. (Table 1).

**Table 1:** MLE, average bias and MSEs of EOLE distribution.

Parameter values		*α* = 0.5, *θ* = 1.0, *λ* = 1.5, *δ* = 1.0			*α* = 2.5, *θ* = 2.0, *λ* = 2.5, *δ* = 1.0		
Parameters	n	MLE	Bias	MSEs	MLE	Bias	MSEs
*α*	50	0.8712	-0.1287	0.2652	0.2920	-0.7079	0.9834
	200	0.7571	-0.2428	0.2441	0.2732	-0.7267	0.9574
	500	0.6517	-0.3483	0.1286	0.2819	-0.7581	0.9455
	750	0.7096	-0.3903	0.1148	0.1209	-0.8791	0.9386
*θ*	50	1.6965	0.2918	10.1400	0.2372	-1.2627	2.9282
	200	1.7918	0.1965	5.9954	0.2275	-1.2724	2.0381
	500	1.6601	0.1601	1.0341	0.2439	-1.2860	1.9289
	750	1.8159	0.1159	0.1943	0.0654	-1.4345	1.2644
*λ*	50	1.4130	-0.4100	1.7804	0.1606	-1.8393	3.5623
	200	1.4805	-0.4750	1.0475	0.1263	-1.8736	3.4641
	500	1.5249	-0.5194	0.2396	0.1267	-1.8932	3.0623
	750	1.5899	-0.5869	0.2277	0.1096	-2.1096	2.7886
*δ*	50	2.9011	-0.4011	20.3715	1.0076	-1.4923	14.5634
	200	1.3166	-1.1833	8.6108	0.8873	-1.6126	10.5828
	500	0.6411	-1.8588	4.3313	0.6477	-1.8522	9.6764
	750	0.5673	-1.9326	4.0774	0.7146	-1.9853	6.5956
Parameter values		*α* = 1.5, *θ* = 1.5, *λ* = 2.0, *δ* = 3.0			*α* = 1.5, *θ* = 2.0, *λ* = 1.5, *δ* = 2.0		
*α*	50	2.7191	0.7190	29.9028	1.6033	0.6033	0.7699
	200	3.3451	0.3450	21.5646	1.4243	0.4243	0.6646
	500	2.5954	0.2954	20.7377	1.6188	0.4188	0.6493
	750	2.8148	0.0814	19.0672	1.4047	0.4047	0.3487
*θ*	50	2.5606	0.0606	0.2102	1.2169	-0.2830	4.0344
	200	2.5193	0.0193	0.1046	1.4164	-0.3835	2.0576
	500	2.3216	-0.1789	0.0578	1.5773	-0.9226	1.7287
	750	2.3722	-0.1977	0.0255	1.9673	-2.4673	1.7180
*λ*	50	2.7600	-0.2399	5.2628	1.2179	-0.5820	1.7154
	200	3.0469	-0.2469	1.3171	1.3851	-0.6148	0.5215
	500	2.4598	-0.5401	0.8365	1.3787	-0.6212	0.1036
	750	2.4022	-0.5977	0.5152	1.2461	-0.7538	0.0435
*δ*	50	3.9810	0.4810	41.5684	8.1752	23.9737	21.4625
	200	3.2986	-0.2013	14.7965	5.3497	11.9746	15.6388
	500	3.2422	-0.5722	13.2541	14.4746	5.6752	14.9338
	750	3.0160	-0.5951	12.3718	26.4737	2.8497	11.7818

### Real data analysis

#### I. Number of deaths per day due to COVID-19 in Nepal

The COVID-19 is a worldwide pandemic of coronavirus disease in 2019 including Nepal. The first COVID case was confirmed on 23 January 2020 and the first death was on 14 May in Nepal. Due to the COVID-19 pandemic, the government has emphasized a nationwide lockdown from March 24, 2020, to July 21, 2020. Following that, the government concentrated its efforts on the PCR test and other health-related initiatives. Every day, the ministry of health and population have been provided the data regarding COVID-19 issues, such as test positive rate, the number of deaths, the number of infected, and many others. During the research period, researchers collected the data daily from 23 January 2019 to 24 December 2019 all over the country. Every day, the ministry of health and the population of Nepal (MOHP) has been reported the data [26]. Among these data, we select the number of deaths to validate the proposed model. A total of 1,808 deaths were recorded in Nepal at the end of 24 December 2020 due to COVID-19 first wave. Every day, on average, 5.4 ≈ 6 people were died due to COVID-19 (from 23 January to 24 December). The summary finding of daily deaths has been presented in the following table (Table 2).

**Table 2:** Descriptive statistic of the number of death due to COVID-19.

Minimum	*Q*_1_	Median	Mean	*Q*_3_	Maximum
0.0	0.0	1.0	4	10	30

To validate the proposed model, at least two deaths occurred every day as consideration for sample data. In the last 153 days, every day, at least two people have died, as reported below [26].

2, 2, 2, 2, 2, 2, 3, 2, 3, 3, 4, 2, 5, 5, 3, 2, 4, 4, 8, 4, 4, 3, 2, 3, 7, 6, 6, 11, 9, 3, 8, 7, 11, 8, 12, 12, 14, 7, 11, 12, 6, 14, 9, 9, 11, 6, 6, 5, 5, 14, 9, 15, 11, 8, 4, 7, 11, 10, 16, 2, 7, 17, 6, 8, 10, 4, 10, 7, 11, 11, 8, 7, 19, 9, 15, 12, 10, 14, 22, 9, 18, 12, 19, 21, 12, 12, 18, 8, 26, 21, 17, 13, 5, 15, 14, 11, 17, 16, 17, 23, 24, 20, 30, 18, 18, 17, 21, 18, 22, 26, 15, 13, 13, 6, 9, 17, 12, 17, 22, 7, 16, 16, 24, 28, 23, 23,19, 25, 29, 21, 9, 13, 16, 10, 17, 20, 23, 14, 12, 11, 15, 9, 18, 14, 13, 6, 16, 12, 11, 7, 3, 5, 5.

To fit the data, we have to check our data set by graphical representation like TTT plot and box plot.

### Total time test plot

**Fig 2:**
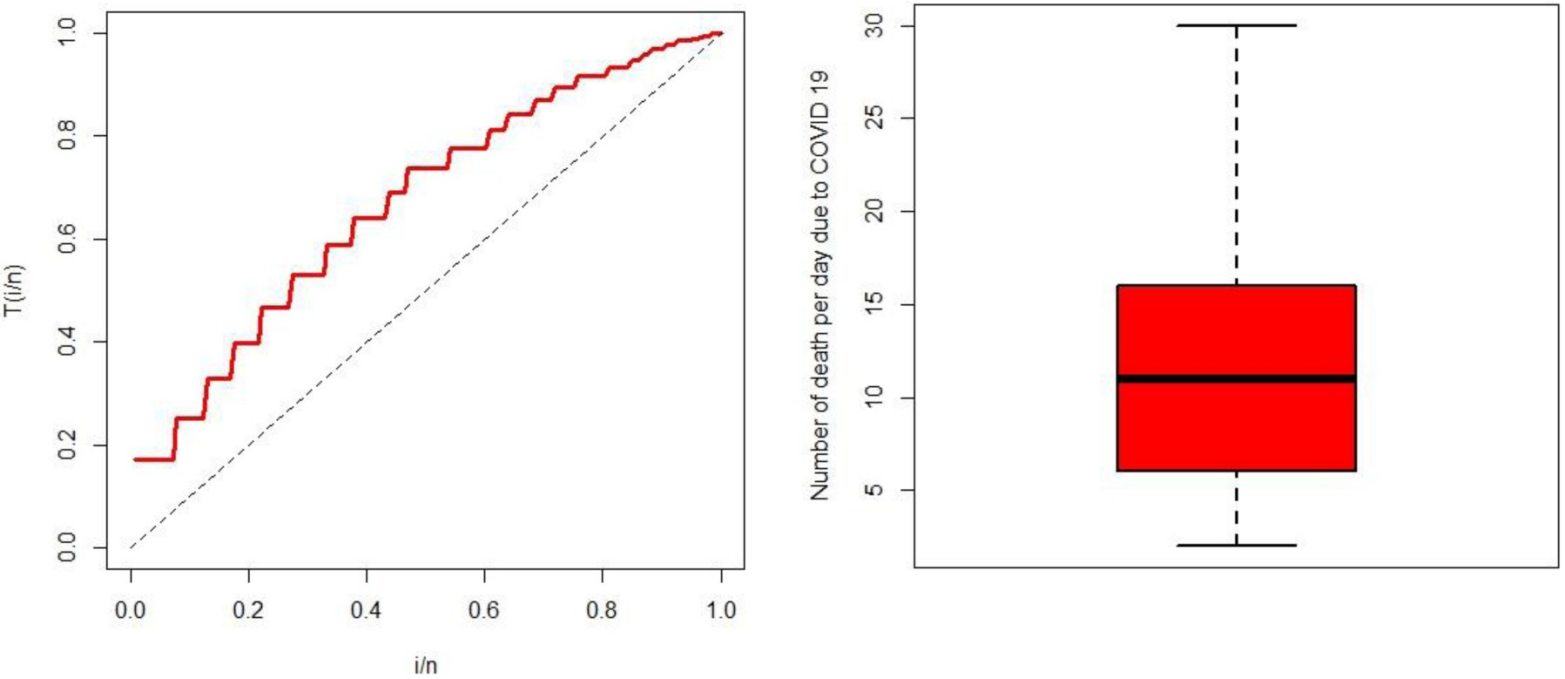
TTT plot (left panel) and box plot (right panel).

TTT plot is an important graphical method for checking whether or not our data set can be applied in a particular model. Plots can be easily obtained by using the TTT function of adequacy model package on R software. It is used to validate the hazard rate function [37, 39]. The empirical version of the TTT plot is

$$T\left(\frac{r}{n}\right)=\frac{\sum_{i=1}^{r}y_{i:n}+(n-r)y_{r:n}}{\sum_{i=1}^{r}y_{i:n}},$$

where, *y*_*r*:*n*_ (*r* = 1, 2, …, *n*) and *y*_*i*:*n*_ (*i* = 1, 2, …, *n*) are the order statistics of the sample. The shape of the TTT plot is either convex for decreasing failure rate, concave for increasing failure rate or bathtub shaped. Here, the TTT plot of the illustrative data set is concave for increasing failure rate. It indicates that the data set is valid for further analysis [Fig 2 (left panel)] [37].

### Box plot

The summary finding of the data set is present by using the box plot. It provides a clear picture of the descriptive characteristics of the illustrative data set [Fig 2 (right panel)].

### Parameter estimation

We computed the value of the parameter by maximizing the log-likelihood function in Eq (28), minimizing the least square method in Eq (35), weighted least square Eq (40), and the Cramér Von Mises method in Eq (45) directly by using *maxLik* () function on R software [38, 39]. Finally, we have to present the estimated value of $\left(\hat{\alpha },\;\hat{\theta },\;\hat{\lambda }\;\text{and}\;\hat{\delta }\right)$; which were computed by different methods (Table 3).

**Table 3:** Estimated parameters’ value from four different methods.

Parameters	MLEs	LSE	WLSE	CVM
$\hat{\alpha }$	0.02665	0.12531	0.04270	0.000392
	(0.0096)	(0.01889)	(0.00137)	(-)
$\hat{\theta }$	1.54569	4.80472	5.09900	1.143173
	(0.12369)	(0.9120)	(0.07107)	(0.4552)
$\hat{\lambda }$	28.60349	0.06219	0.13710	1.007677
	(4.99647)	(0.0025)	(0.00250)	(0.25886)
$\hat{\delta }$	7.52539	0.00063	0.0000018	0.001703
	(5.2967)	(-)	(-)	(0.02582)

Standard error in parenthesis.

### Distribution characteristics

After estimating the value of the parameter, we determined the characteristics of the proposed distribution from the illustrative data set. The finding of descriptive statistics showed that the mean is greater than the median, which is also higher than the mode, and value of skewness is positive, which shows that the proposed model is positively skewed. In the case of kurtosis, the distribution is approximately symmetrical, but towards platykurtic (Table 4).

**Table 4:** Descriptive characteristic of the proposed model.

Mean	Median	Mode	Standard deviation	Skewness	Kurtosis
0.03148474	0.02592923	0.07018629	0.01871639	0.7133013	2.363588

### Validation of estimation methods

Various methods have been used in the literature to estimate unknown parameters. Among them, we used four methods named: MLE, LSE, WLSE, and CVM. Again, we have to check the validation of the different methods by using different goodness of fit criteria. The well-known criteria are Kolmogorov-Simnorov (KS) test, Anderson’s darling (*A*^2^) test, and Cramér Von Mises (*W*) test. The p-values of the KS test, *A*^2^ test, and *W* test are insignificant with finding of MLE, but significant with the finding of LSE, WLSE, and CVM. Therefore, MLE has satisfied the good behavior of goodness of fit (Table 5).

**Table 5:** Comparison with a p-value of KS, *A*^2^ and *W* statistics in different methods.

Methods	KS (p-value)	*A*^2^(p-value)	*W*(p-value)
MLE	0.044551 (0.5448)	0.52371(0.7225)	0.060119(0.814)
LSE	0.17277(0.0001079)	13.388(3.922×10^−6^)	2.2771(2.67×10^−6^)
WLSE	0.37789(2.2×10^−16^)	35.44(3.922×10^−6^)	7.5955(2.2×10^−16^)
CVM	0.15457(0.0006679)	13.196(3.922×10^−6^)	2.1222(6.064×10^−6^)

**Fig 3:**
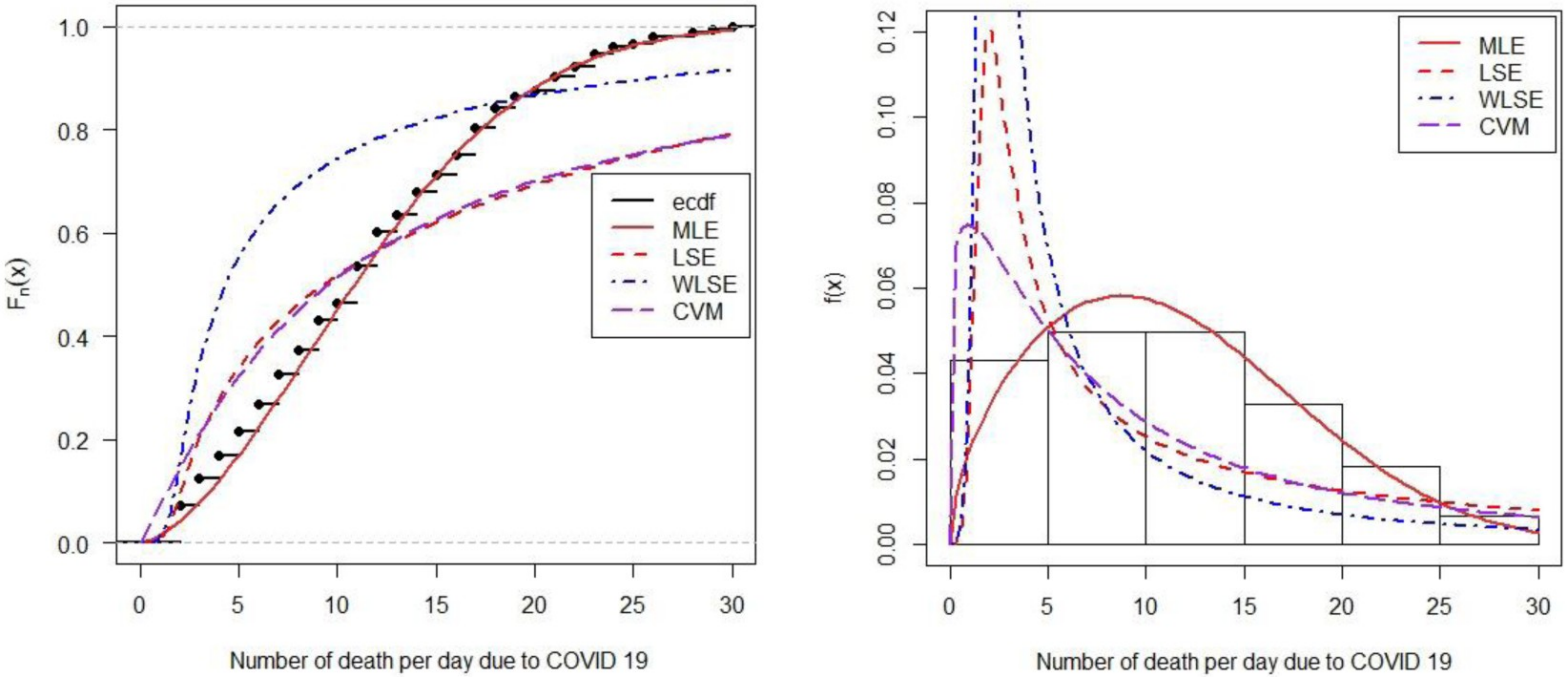
Plot the empirical distribution function with estimated CDF (left panel), histogram with estimated PDF (right panel).

Furthermore, we compared the empirical distribution and theoretical cumulative distribution of the proposed model, indicating that the curve of empirical distribution is closer with the finding of MLE but does not closer with other findings (LSE, WLSE, and CVM) in the illustrative data set [Fig 3 (left panel)]. Also, we plot the theoretical PDF of the intended model by using different estimated values [Fig 3 (right panel)]. In both graphical demonstrations, the estimated value of MLE is more appropriately fitted than others.

### Relationship between the predictive probability of number of deaths and test Positive rate

Again, we have to estimate the parameter value of EOLE distribution by using the test positive rate per day from the MLE technique. The estimated parameter’s value of EOLE distribution with Standard Error (SE) are $\left(\hat{\alpha }=0.0676\;(0.035)\right)$; $\left(\hat{\theta }=2.726\left(0.410\right)\right)$; $\left(\hat{\lambda }=1.556\left(0.514\right)\right)$ and, $\left(\hat{\delta }=1.105\;\left(2.113\right)\right)$.

Furthermore, we have to predict the probability of test positive rate and probability of number of deaths per day. Finally, we have to determine the relationship among these variables. The finding revealed that, there is a positive relationship among these variables, which is statistically significant (r = 0.2762, p-value = 0.00054) with a 95% confidence interval (0.12291–0.41662).

**Fig 4:**
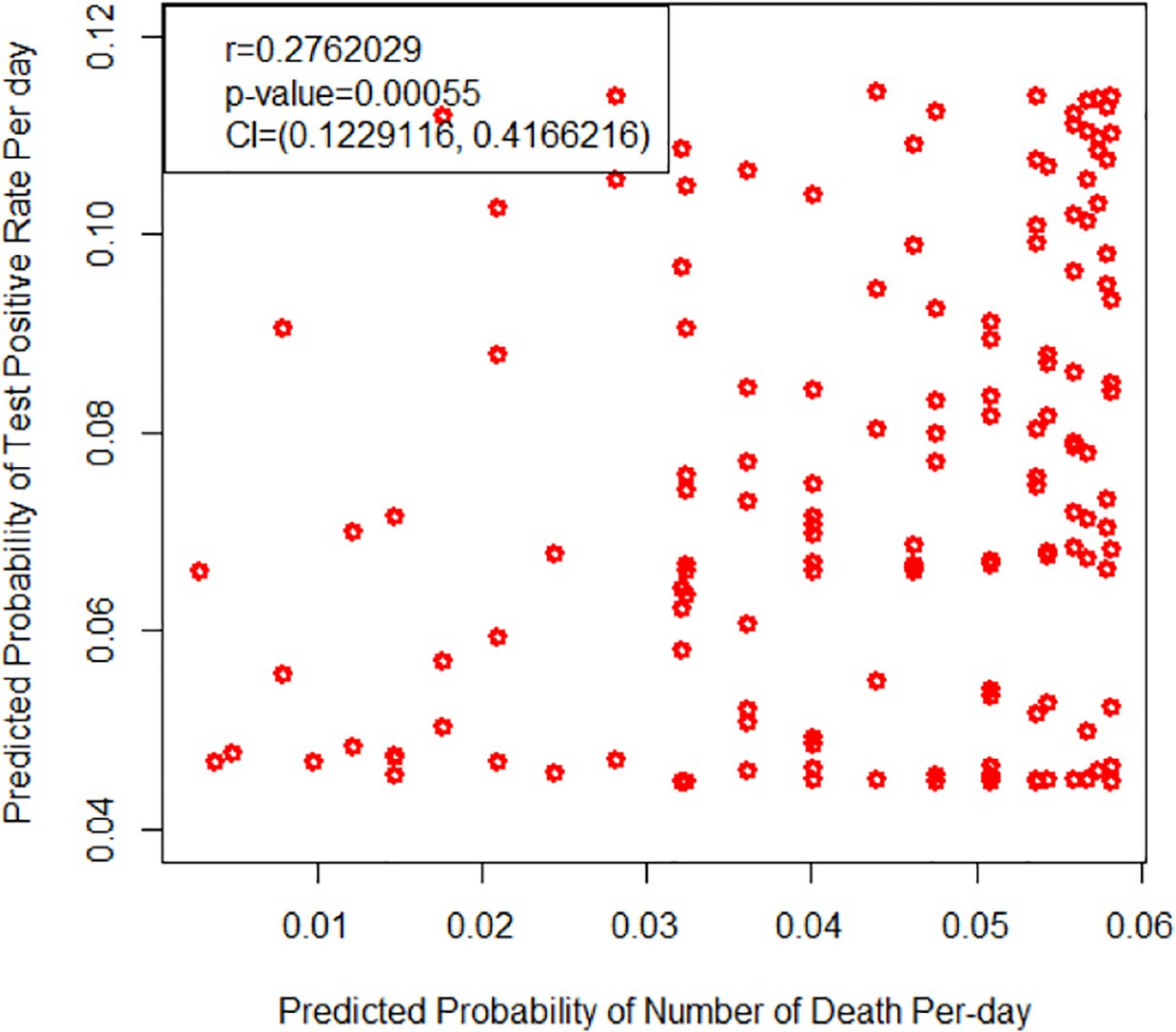
Relationship between predictive prob-ability of number of death and test positive rate.

The finding concludes that the test positive rate will increase; the death rate should be increased [Fig 4].

### Model comparisons/selections

Model selection is an important and integral part of data analysis. It is important to increase computing power to fit more realistic, flexible, and complex models. We compared our proposed model with eleven competitive models namely; exponentiated half logistic exponential (EHLE) [40], Marshall-Olkin logistic exponential (MOLE) [41], Lomax exponential Weibull (LEW) [42], exponentiated generalized inverted exponential (EGIE) [43], generalized inverted generalized exponential (GIGE) [44], generalized odd inverted exponential exponential (GOIEE) [45], Marshall–Olkin power generalized Weibull (MOPGW) [46], odd Lomax exponential (OLE) [47], type I half-logistic Fréchet (TIHLF) [48], Lindley inverse Weibull (LIW) [36] and half logistic Nadarajah Haghighi extension of exponential (HLNHE) [19]. To compare the proposed models with other competitive models, firstly we determine the value of parameters by *maxlik function ()* from R software by solving the nonlinear equation [38, 39]. The estimated parameter value of each distribution along with standard error are present in the following table (Table 6). *The PDF of each competitive model is in Appendix C of* S1 Appendix.

**Table 6:** Estimated value of parameters: Proposed as well as competitive models.

Models	$\hat{\alpha }$	$\hat{\beta }$	$\hat{\lambda }$	$\hat{\delta }$	$\hat{\gamma }$	$\hat{\theta }$
EOLE	0.02665	-	28.60349	7.52539	-	1.54569
	(0.0096)		(4.9964)	(5.296)		(0.0096)
EHLE	0.09536	1.90552	1.75018	-	-	-
	(0.40547)	(0.2216)	(7.4483)			
MOLE	1.28741	-	0.14677	-	-	5.58735
	(0.2403)		(0.0449)			(3.0400)
LEW	6.89946	1.05636	-	-	-	0.085955
	(0.00049)	(-)				(0.00695)
EGIE	7.62274	0.23071	44.14876	-	-	-
	(-)	(0.01863)	(-)			
GIGE	2.3108	-	4.8503	-	2.4017	-
	(0.3062)		(-)		(-)	
GOIEE	-	-	0.61419	-	4.31244	0.15720
			(0.1901)		(2.0892)	(0.0137)
MOPGW	0.78360	1.91966	0.01048	-	-	-
	(0.2434)	(0.2191)	(0.0037)			
OLE	3.64656	14.66365	-	-	-	0.12644
	(0.9066)	(4.1751)				(0.0187)
TIHLF	111.25627	0.46544	23.3395	-	-	-
	(2.6417)	(0.0149)	(1.9467)			
LIW	8.6377	0.7461	-	-	-	4.1896
	(0.8695)	(0.1045)				(1.4514)
HLNHE	0.04697	1.63999	1.33342	-	-	-
	(-)	(0.2991)	(-)			

Standard error in parenthesis.

We have compared different goodness of fit criteria like as; (i) values of log-likelihood, (ii) Akaike’s information criterion, (iii) Bayesian information criterion, (iv) corrected Akaike’s information criterion, and (v) Hannan-Quinn information criterion. Each criteria can be calculated as following relation; $\text{AIC}=-2l(\hat{\theta })+2p$; $\text{BIC}=-2l(\hat{\theta })+pln(n)$; $\text{CAIC}=AIC+\frac{2p(p+1)}{n-p-1}$; and $HQIC=-2l(\hat{\theta })+2pln(ln(n))$. Where, *p* is the number of parameters in the model and *n* is the total sample under consideration.

According to -2LL, AIC, BIC, CIAC and HQIC, the least value among the competitive models is superior to others. The finding reveals that the value of the intended model has smaller as compared to all other eleven competitive models. Therefore, the proposed model is superior than others followed by MOPGW. The model GIGE is the least fitted model in the given illustrative data set (Table 7).

**Table 7:** Calculated value of -2LL, AIC, BIC, CIAC, and HQIC of different models.

Models	$l(\hat{\theta })$	-2LL	AIC	BIC	CIAC	HQIC
EOLE	-496.8049	993.6098	1001.609	1013.732	1001.879	1006.534
EHLE	-499.7089	999.4178	1005.418	1014.509	1005.579	1009.111
MOLE	-499.7126	999.4252	1005.425	1014.517	1005.586	1009.118
LEW	-512.8346	1025.669	1031.669	1040.761	1031.830	1035.362
EGIE	-507.7668	1015.534	1021.534	1030.625	1021.695	1025.227
GIGE	-520.2300	1040.460	1046.460	1055.551	1046.621	1050.153
GOIEE	-498.4237	996.8474	1002.847	1011.939	1003.008	1006.540
MOPGW	-497.9371	995.8742	1001.874	1010.966	1002.035	1005.567
OLE	-499.5843	999.1686	1005.169	1014.260	1005.330	1008.862
TIHLF	-502.0950	1004.190	1010.190	1019.281	1010.351	1013.883
LIW	-514.9643	1029.929	1035.929	1045.020	1036.090	1039.622
HLNHE	-506.3778	1012.756	1018.756	1027.847	1018.917	1022.449

**Fig 5:**
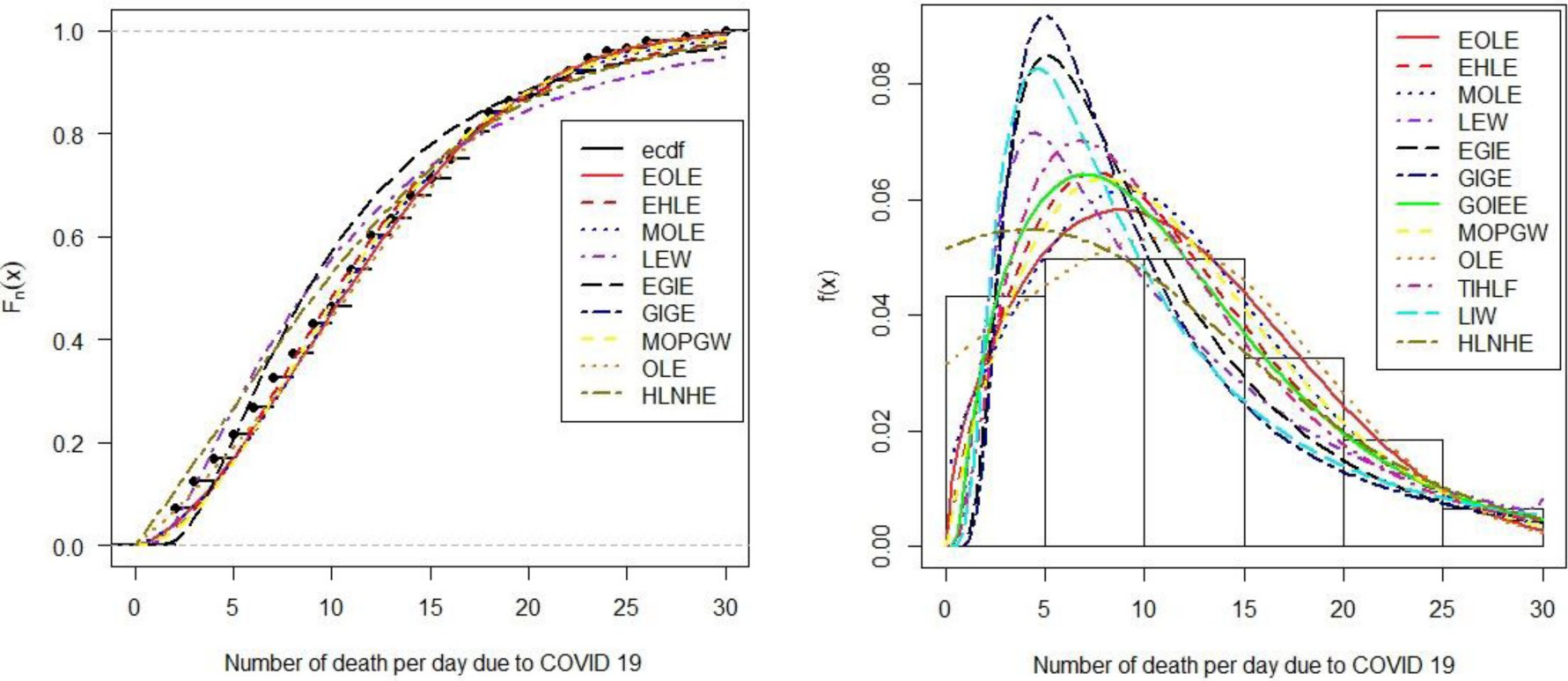
Estimated fitted CDF (left panel), Estimated fitted densities (right panel).

Furthermore, we have compared the empirical distribution and theoretical cumulative distribution of the proposed model, indicating that both curves are closer in the illustrative data set. Likewise, the theoretical CDF of nine competitive models namely, EHLE, MOLE, LEW, EGIE, GIGE, MOPGW, OLE, LIW, and HLNHE compared to the theoretical CDF the proposed model [Fig 5 (left panel)]. Also, the theoretical PDF of the intended model is compared with all other competitive models [Fig 5 (right panel)]. The finding suggests that the proposed model is adequately fit in illustrative data set than all other competitive models.

#### II. Failure stresses (In Gpa) of single carbon fibers of lengths 50 mm data set

The second data set “on failure stresses (in GPa) of single carbon fibers of lengths 50 mm” [49] has been used to validate the proposed distribution. The illustrative data set were used by different authors to validate other distributions like, a new extension of the generalized half logistic distribution [50] and weighted Lindley distribution [51].

1.339, 1.434, 1.549, 1.574, 1.589, 1.613, 1.746, 1.753, 1.764, 1.807, 1.812, 1.84, 1.852, 1.852, 1.862, 1.864,1.931, 1.952, 1.974, 2.019, 2.051, 2.055, 2.058, 2.088, 2.125, 2.162, 2.171, 2.172, 2.18, 2.194, 2.211, 2.27, 2.272,2.28, 2.299, 2.308, 2.335, 2.349, 2.356, 2.386, 2.39, 2.41, 2.43, 2.431, 2.458, 2.471, 2.497, 2.514, 2.558, 2.577, 2.593, 2.601, 2.604, 2.62, 2.633, 2.67, 2.682, 2.699, 2.705, 2.735, 2.785, 3.02, 3.042, 3.116, 3.174.

Now, we have used an illustrative data set to estimate the parameters value of the proposed model. The estimated value of the parameters $\left(\hat{\alpha },\;\hat{\theta },\;\hat{\lambda }\;\text{and}\;\hat{\delta }\right)$ are (0.120098, 6.208652, 3.391613 and 0.003138) respectively. Furthermore, we used the KS test, Anderson’s darling test (*A*^2^), and Cramér Von Mises test (*W*) to assess the goodness of fit. The test values for each statistic are 0.038742 (p-value = 0.8227), 0.21115 (p-value = 0.9871), and 0.025715 (p-value = 0.9886), respectively. The p-values of each statistic support the null hypothesis, indicating that the proposed model has a better fit in the recommended data set. Similarly, we compared the proposed model to other competitive models using -2LL, AIC, BIC, CIAC, and HQIC. Firstly, we estimate the values of the model’s parameters and present them in the table (Table 8).

**Table 8:** Estimated parameter values of all competitive models.

Models	$\hat{\alpha }$	$\hat{\beta }$	$\hat{\lambda }$	$\hat{\delta }$	$\hat{\gamma }$	$\hat{\theta }$
EOLE	0.1201	-	3.3916	0.00314	-	6.2086
	(0.08131)		(4.7154)	(0.0122)		(1.0895)
EHLE	1.172	88.795	2.168	-	-	-
	(8.931)	(27.318)	(16.395)			
MOLE	5.7126	-	0.4646	-	-	30.9984
	(0.7186)		(0.0318)			(8.7419)
GOIEE	-	-	11.8619	-	14.1011	2.5457
			(-)		(-)	(0.1892)
MOPGW	1.5665	4.1652	0.01511	-	-	-
	(0.5538)	(0.4992)	(0.0071)			
OLE	1.7423	249.616	2.2136	-	-	-
	(0.5431)	(-)	(0.1738)			
HLNHE	1.3485	3.4176	0.0118	-	-	-
	(0.32677)	(0.41701)	(0.01005)			

Standard error in parenthesis.

The lowest value of -2LL, AIC, BIC, CIAC, and HQIC in the proposed model, among all competitive models, indicates that the proposed model is superior to others (Table 9).

**Table 9:** Comparison of -2LL, AIC, BIC, CIAC, and HQIC value among models.

Models	$l(\hat{\theta })$	-2LL	AIC	BIC	CIAC	HQIC
EOLE	-35.05741	70.1148	78.1148	86.8123	78.78146	80.5465
EHLE	-38.3148	76.6296	82.6296	89.1527	83.0230426	85.2034
MOLE	-36.0807	72.1614	78.1609	84.6845	78.5543426	80.7352
GOIEE	-38.3202	76.6404	82.6403	89.1635	83.0337426	85.2142
MOPGW	-39.1957	78.3914	84.3914	90.9145	84.7848426	86.9652
OLE	-44.9061	89.8122	95.8123	102.335	96.2057426	98.386
HLNHE	-53.7108	107.422	113.4216	119.945	113.815043	115.995

**Fig 6:**
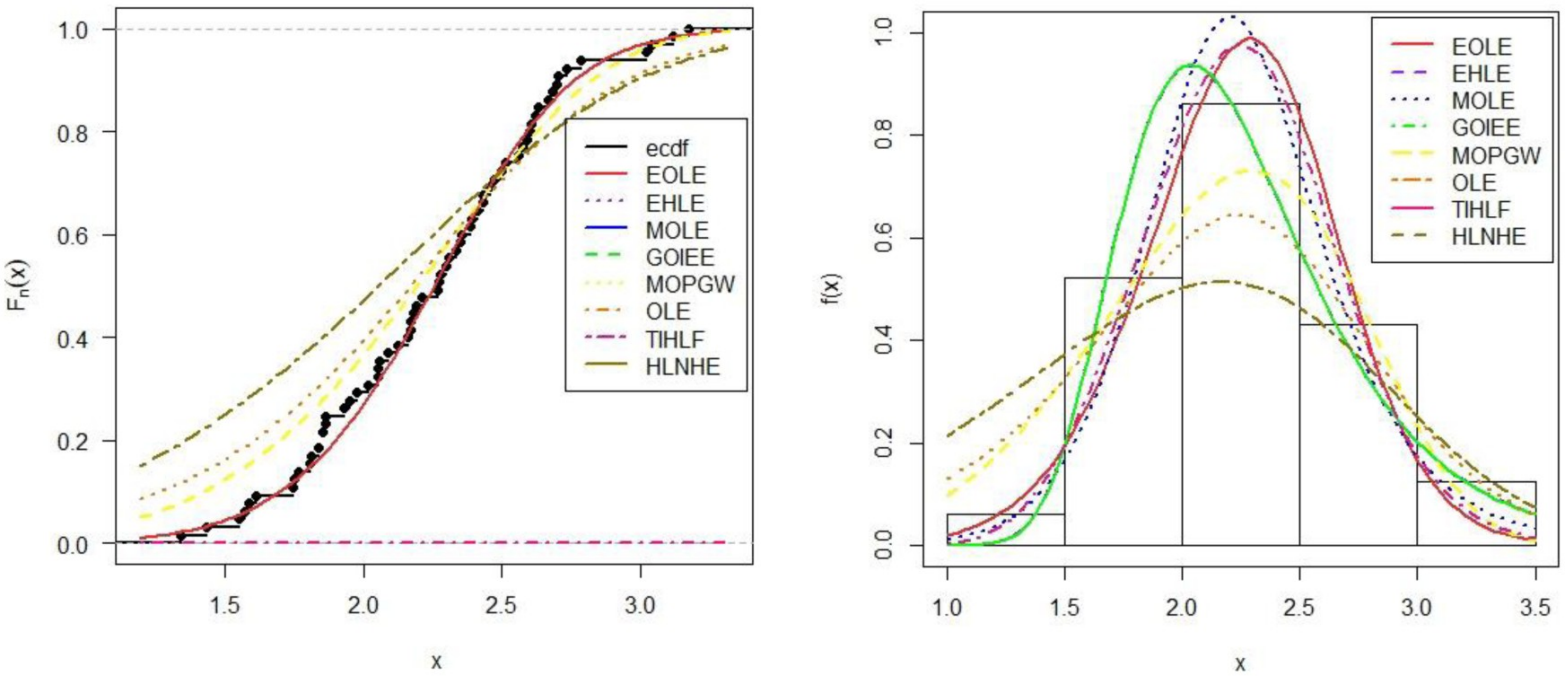
Estimated fitted CDF (left panel), Estimated fitted densities (right panel) of carbon fiber data set.

Similarly, the built model is appropriately fit in terms of graphical appearance than other competitive models [Fig 6].

## Conclusion

This study suggested a new four-parameter Exponentiated Odd Lomax Exponential (EOLE) distribution by compounding an exponentiated odd function with Lomax distribution as a generator. Some important properties of the new distribution are investigated such as quintile function and median; asymptotic properties and mode; moments; mean residual life, mean path time; mean deviation; order statistics; and Bonferroni & Lorenz curve. Further, we have employed three well-known estimation methods to estimate the model parameters namely, the maximum likelihood estimation, least-square estimation, and Cramér-Von-Mises methods. To verified the different theoretical finding we have applied a simulation study and two real data sets, ‘‘Number of deaths per day due to COVID-19 first wave in Nepal” and ‘‘failure stresses (in GPa) of single carbon fibers of lengths 50 mm”. It has a significantly positive relationship between predicted test positive rate and the predicted number of deaths per day. Finally, we analyzed the illustrative data set and found that the proposed model provides a reasonably better fit as compared to some other well-known models. Therefore, the EOLE distribution can be used as an alternative model in the future to analyze survival and lifetime data.

## Supporting information

S1 Appendix (DOCX)
