# Supplementary material for: Exponentiated Odd Lomax Exponential distribution with application to COVID-19 death cases of Nepal
Source: PLoS One. 2022 Jun 3;17(6):e0269450. doi: 10.1371/journal.pone.0269450 (PMC9165905; doi:10.1371/journal.pone.0269450)
Supplement: S1 Appendix — (DOCX) [file pone.0269450.s001.docx]

**Appendix A**

**Preposition 1:** The PDF of the proposed distribution can be derived as;

$$f(x)=\frac{\alpha\theta\lambda}{\delta}e^{\alpha x}(e^{\alpha x}-1)^{\theta-1}\left[1+\frac{1}{\delta}\left\{e^{\alpha x}-1\right\}^{\theta}\right]^{-(\lambda+1)}$$

= $\frac{\alpha\theta\lambda}{\delta^{i+1}}e^{\alpha x}(e^{\alpha x}-1)^{\theta-1}\sum_{i=0}^{\infty} (-1)^{i}\left(\begin{matrix} \lambda+i \\ i \end{matrix}\right)\left(e^{\alpha x}-1\right)^{\theta i}$

= $\frac{\alpha\theta\lambda}{\delta^{i+1}}e^{\alpha x}\sum_{i=0}^{\infty} (-1)^{i}\left(\begin{matrix} \lambda+i \\ i \end{matrix}\right)\left(e^{\alpha x}-1\right)^{\left\{\theta(i+1)-1\right\}}$

= $\frac{\alpha\theta\lambda}{\delta^{i+1}}e^{\alpha x}\sum_{i=0}^{\infty} (-1)^{i+\theta(i+1)-1}\left(\begin{matrix} \lambda+i \\ i \end{matrix}\right)\left(1-e^{\alpha x}\right)^{\left\{\theta(i+1)-1\right\}}$

= $\frac{\alpha\theta\lambda}{\delta^{i+1}}\sum_{i=0}^{\infty} \sum_{j=0}^{\infty} (-1)^{i+\theta(i+1)+j-1}\left(\begin{matrix} \lambda+i \\ i \end{matrix}\right)\left(\begin{matrix} \theta(i+1)-1 \\ j \end{matrix}\right)e^{\alpha(1+j)x}$

=$\sum_{i=0}^{\infty} \sum_{j=0}^{\infty} \Phi_{ij}e^{\alpha(1+j)x}$; where, $\Phi_{ij}=\frac{\alpha\theta\lambda}{\delta^{i+1}}(-1)^{i+\theta(i+1)+j-1}\left(\begin{matrix} \lambda+i \\ i \end{matrix}\right)\left(\begin{matrix} \theta(i+1)-1 \\ j \end{matrix}\right).$

**Preposition 2:** The CDF of proposed distribution is also derived as the expansion of generalization of binomial series;

$$F\left(x\right)=1-\left[1+\frac{1}{\delta}\left\{e^{\alpha x}-1\right\}^{\theta}\right]^{-\lambda}$$

**=$1-\sum_{i=0}^{\infty} \frac{\left(-1\right)^{i}}{\delta^{i}}\left(\begin{matrix} \lambda+i-1 \\ i \end{matrix}\right)\left(e^{\alpha x}-1\right)^{\theta i}$**

**=$1-\sum_{i=0}^{\infty} \sum_{j=0}^{\infty} \frac{(-1)^{(\theta+1)i+j}}{\delta^{i}}\left(\begin{matrix} \lambda+i-1 \\ i \end{matrix}\right)\left(\begin{matrix} \theta i \\ j \end{matrix}\right)e^{\alpha jx}$**

=$1-\sum_{i=0}^{\infty} \sum_{j=0}^{\infty} \eta_{ij}e^{\alpha jx}$; where, $\eta_{ij}=\frac{(-1)^{(\theta+1)i+j}}{\delta^{i}}\left(\begin{matrix} \lambda+i-1 \\ i \end{matrix}\right)\left(\begin{matrix} \theta i \\ j \end{matrix}\right).$

**Appendix B**

Element of observed Information Matrix

 (1)

 (2)

 (3)

 (4)

 (5)

 (6)

 (7)

 (8)

 (9)

 (10)

**Appendix C**

Probability Density Function of competitive models which are compared with EOLE distribution

$f_{EHLE}(x)=\frac{2a\lambda\alpha e^{-\alpha\lambda x}[1-e^{-\alpha\lambda x}]^{a-1}}{[1+e^{-\alpha\lambda x}]^{a+1}};x>0,\mathrm{a}>0,\alpha>0,\lambda>0.$ (1)

$f_{MOLE}(x)=\frac{\alpha\theta\lambda e^{\lambda x}(e^{\lambda x}-1)^{-\alpha-1}}{[1+\theta(e^{\lambda x}-1)^{-\alpha}]^{2}};x>0,\alpha>0,\theta>0,\lambda>0.$ (2)

$f_{EGIE}(x)=\alpha\beta\lambda x^{-2}e^{-\lambda/x}(1-e^{-\lambda/x})^{\alpha-1}\left[1-(1-e^{-\lambda/x})^{\alpha}\right]^{\beta};x>0,\alpha>0,\lambda>0,\gamma>0.$ (3)

$f_{GIGE}(x)=\alpha\lambda\gamma x^{-2}e^{-\gamma(\lambda/x)}(1-e^{-\gamma(\lambda/x)})^{\alpha-1};x>0,\alpha>0,\lambda>0,\gamma>0.$ (4)

$f_{OLE}(x)=\alpha\beta^{\alpha}\lambda e^{\lambda x}\left[\beta+\left(e^{\lambda x}-1\right)\right]^{-(\alpha+1)};x>0,\alpha>0,\beta>0,\lambda>0.$ (5)

$f_{TIHLF}(x)=\frac{2\lambda\beta\alpha^{\beta}x^{-\beta-1}\left[1-e^{-(\alpha/x)^{\beta}}\right]^{\lambda-1}}{\left\{1+\left[1-e^{-(\alpha/x)^{\beta}}\right]^{\lambda}\right\}^{2}};x>0,\alpha>0,\beta>0,\lambda>0.$ (6)

$f_{HLNHE}(x)=\frac{2\alpha\beta\lambda(1+\alpha x)^{(\beta-1)}e^{\lambda\left[1-(1+\alpha x)^{\beta}\right]}}{\left[1+e^{\lambda\left[1-(1+\alpha x)^{\beta}\right]}\right]^{2}};x>0,\alpha>0,\beta>0,\lambda>0.$ (7)$\begin{matrix} f_{LEW}(x)=\frac{\alpha\beta\theta x^{\beta-1}e^{-x^{\beta}}\left(1-e^{-x^{\beta}}\right)^{\alpha-1}}{\left[1-\left(1-e^{-x^{\beta}}\right)^{\alpha}\right]^{2}}\left\{1+\frac{\left(1-e^{-x^{\beta}}\right)^{\alpha}}{1-\left(1-e^{-x^{\beta}}\right)^{\alpha}}\right\}^{-(\theta+1)}; \\ \text{ }x\geq0,\alpha\geq0,\beta\geq0,\theta\geq0 \end{matrix}$ (8)

$f_{GOIEE}(x)=\frac{\theta\lambda\left[1+\gamma\left(1-e^{-\theta x}\right)^{2}\right]}{\left(1-e^{-\theta x}\right)^{2}}e^{-\text{ }\frac{\lambda\left[1+\gamma\left(1-e^{-\theta x}\right)\right]}{e^{\theta x}-1}};x\geq0,\gamma\geq0,\lambda\geq0,\theta\geq0$ (9)

$f_{MOPGW}(x)=\alpha\beta\lambda x^{\beta-1}(1+\lambda x^{\beta})^{\alpha-1}\mathrm{e}^{1-(1+\lambda x^{\beta})^{\alpha}};x>0,\alpha>0,\beta>0,\lambda>0.$ (10)

$\begin{matrix} f_{LIW}(x)=\alpha\beta\left(\frac{\theta^{2}}{\theta+1}\right)x^{-(\beta+1)}\mathrm{e}^{-\alpha x^{-\beta}}\left(1-\mathrm{e}^{-\alpha x^{-\beta}}\right)^{\theta-1}\left\{1-\ln\left(1-\mathrm{e}^{-\alpha x^{-\beta}}\right)\right\}; \\ \text{ }x>0,\alpha>0,\beta>0,\theta>0. \end{matrix}$ (11)
